# *Leishmania infantum* LeIF protein is an eIF4A-like RNA helicase that modulates interleukin IL-12p70, IL-10 and TNF-α production in human monocytes

**Published:** 2011-01-10

**Authors:** Mourad Barhoumi, N Kyle Tanner, Amel Garnaoui, Josette Banroques, Belhassen Kaabi, Patrick Linder, Ikram Guizani

**Affiliations:** 1 Laboratoire d’Epidémiologie et d’Ecologie Parasitaire, Institut Pasteur de Tunis, 1002 Tunis-Belvedère, Tunisia; 2 Département de Microbiologie et Médicine Moléculaire, Centre Médical Universitaire, Genève, Switzerland; 3 Laboratoire d’Immunologie, Vaccinologie et Génétique Moléculaire, Institut Pasteur de Tunis, 1002 Tunis-Belvedère, Tunisia; 4 Centre de Génétique Moléculaire, CNRS, Gif-sur-Yvette, France

## 

*Leishmania* LeIF antigen, homologous to eukaryotic initiation factor eIF4A, was originally described as a Th1-type natural adjuvant and as an antigen that induces an IL-12-mediated Th1 response in the peripheral blood mononuclear cells (PBMC) of leishmaniasis patients. We aimed at addressing the role of this protein and defining the minimum fragment necessary for inducing cytokine secretion. The study necessitated expression cloning of *LeIF* and 9 domains. Comparative biochemical and genetic analyses of LeIF and yeast eIF4A showed that LieIF is both an RNA-dependent ATPase and ATP-dependent RNA helicase in vitro and highlighted differences with yeast protein. *In vivo* experiments in yeast showed that *LeIF* cannot complement the deletion of the essential *TIF1* and *TIF2* genes in the yeast *Saccharomyces cerevisiae* that encode eIF4A. However, expression of LeIF results in a dominant negative phenotype, which is abolished by deletion of the most divergent 25 N-terminal residues. LeIF is able to interact with yeast eIF4G; this suggested a role in the translation machinery. The assays measuring production of cytokines IL-12p70, IL-10 and TNF-α by PBMC-derived monocytes of healthy donors exposed to the different proteins showed that LieIF was able to induce the secretion of these cytokines. Unlike previous reports on LeIF from *L. braziliensis* and *L. major*, both amino and carboxyl parts of the protein were shown to induce the secretion of cytokines at significant levels. Our results suggest that this activity could be primarily located in amino acids 1-129 and 261-403. Furthermore, the induction of cytokines in monocytes of healthy subjects is not unique to the *Leishmania* protein. Indeed, 5 homologous proteins DEAD box in mammals and yeast were also able to induce the secretion of cytokines. This study confirms the importance of LeIF protein as a vaccine target and underscores its potential as drug target.

